# Editorial: Stimuli-responsive smart materials for biomedical applications

**DOI:** 10.3389/fbioe.2022.1020923

**Published:** 2022-09-13

**Authors:** Jie Ju, I-Chi Lee, Yi-Chen Ethan Li

**Affiliations:** ^1^ Key Laboratory for Special Functional Materials, School of Materials, Henan University, Henan, China; ^2^ Department of Biomedical Engineering and Environmental Sciences, National Tsing Hua University, Hsinchu, Taiwan; ^3^ Department of Chemical Engineering, Feng Chia University, Taichung, Taiwan

**Keywords:** stimuli-responsive, smart materials, tissue engineering, regenerative medicine, drug controlled release system, personalized medicine

Recently, stimuli-responsive biomaterials enable responding to the changes in various environmental signals and have increased attention to developing a new type of materials for fabricating a dynamic system (Municoy et al., 2020). This special issue summarizes the current trends in the development of stimuli-responsive biomaterials for the creation of reliable biomimetic systems that recapture the response to the physiological environment under the changes in pH, magnetic field, electrical field, ionic strength, light, and heat.

The human body is a dynamic living system that can rapidly respond to changes in the physiological environment. Therefore, recapitulating the dynamic behaviors of tissues/organs *in vitro* plays an indispensable role in modeling biomimetic systems. In the past few decades, stimuli-responsive material-based *in vitro* systems have brought a bridge between the static and dynamic artificial biological systems, which provides a new idea to develop versatile and dynamic biomimetic cell-based systems via the control of stimuli ranging from external and internal triggers (Khademhosseiniand Langer 2016). Therefore, stimuli-responsive biomaterial-based systems promise to design smart biomimetic systems with functions or structures to obtain reliable results from *in vitro* testing similar to the *in vivo* biological equivalents (Mura et al., 2013). Based on this information, stimuli-responsive materials provide a strategy to fabricate new-type dynamic systems and benefit the applications in the field of tissue engineering, organ-on-chips, drug testing/drug repurposing, and drug control delivery for translation medicine.

Luo and co-workers developed a theranostics platform composed of a chemotherapeutic agent, superparamagnetic iron oxide, and manganese dioxide to overcome the limitation of using a single chemotherapeutic strategy and a single diagnostic imaging mode in the applications of cancer precision medicine. Furthermore, with the results from *in vitro* and *in vivo* experiments, the hybrid nanoparticles as the platform can achieve efficient magnetic resonance imaging and be an inducer for offering the synergistic effect of chemotherapy and magnetic hyperthermia on the inhibition of tumor growth. The results confirmed that smart nanoparticle-based probes have a promising potential for tumor diagnosis and therapy.

Du and co-workers focused on developing hemostatic containing an antioxidant and antimicrobial function to reduce the mortality caused by uncontrolled blood loss and microbial infections. In the study, they successfully synthesized phenolic acid-based shape memory polyurethane foams, which can be stably stored under the ambient environment and rapid swell in water at physiological temperature. Furthermore, the shape memory foams with phenolic acid significantly the effect of antimicrobial and antioxidant, providing a useful system for reducing microbial infections and bleeding-related mortality and improving the outcome of wound healing.

Su and co-workers developed acid-responsive nano-level micelles as a pharmaceutic carrier for the treatment of osteoarthritis. In their study, a hydrazone-linkage-based pH-sensitive methoxy poly (ethylene oxide)-hydrazone-poly (ε-caprolactone) (mPEG-Hz-b-PCL) polymer was synthesized and used as a drug delivery system. Furthermore, the mPEG-Hz-b-PCL nanomicelles encapsulated a chondrogenesis-induced small molecule and was evaluated the effects of small molecule-laden nanomicelles on the treatment of osteoarthritis.

Kao et al. reported using an implant coating with a smart drug release effect to reduce the risk of bacterial infection from the incompletely sterilized surgery equipment and unclean medical environment. Furthermore, they coated a polyelectrolyte multilayer containing the composite with mesoporous bioactive glass and trace elements on the metal materials and discussed the effect of the coating on the applications in medical dentistry and orthopedics. Moreover, according to the results from *in vitro* and *in vivo* experiments, the technology of coating polyelectrolyte multilayer with a smart release of trace elements offers functions to promote antibacterial activity, osseointegration, and angiogenesis for medical dentistry and orthopedics applications.

Ghorbani and co-workers proposed injectable sodium humate-laden methylcellulose hydrogels with light-assisted thermoresponsive properties to control the release of chemotherapy agents. Subsequently, they discussed the effect of sodium humate on the regulation of mechanical properties and durability of hydrogels and the synergistic effect of hydrogels’ photo-heat transition and drug release behaviors on osteosarcoma cell viability. The results show that the feasibility and potential of the stimuli-responsive drug delivery systems with simultaneous photothermal and chemotherapy functions set the foundation for future preclinical and clinical trials.

In conclusion, we appreciated the contributors to their research expertise and discussed the applications of stimuli-responsive biomaterials for making biomimetic dynamic systems to obtain reliable results from *in vitro* and *in vivo* experiments. We hope this special issue will provide new strategies to create versatile and innovative stimuli-responsive biomaterials for tissue engineering, drug control release, diagnosis, and personalized medicine.
